# Meetings of Societies

**Published:** 1946

**Authors:** 


### Meetings of Societies

### Bristol Medico-Chirurgical Society

The Annual General Meeting of this Society was held at the Physics Lecture Theatre of the University on Wednesday, 9th October, at 8.15 p.m.

Dr. H. H. Carleton was in the Chair, and eighty-six members and guests were present :—

1. After the minutes were read and approved, the Secretary read a letter from Dr. Maarschalk of Arnhem, suggesting interchange of visits between medical men of Bristol and Arnhem.

2. The nature of the forthcoming Clinical meeting was then discussed. It was decided to show about eight cases from the stage in the course of the evening, and to limit the discussion so as to get through this programme in one and a half hours.

3. Dr. Carleton then resigned his chair to the new President, Mr. H. Chitty.

A vote of thanks to the retiring President was proposed by Dr. Orr-Ewing and seconded by Mr. Duncan Wood. The new President then gave his presidential address entitled "Back to Work." Mr. Chitty covered the whole problem of returning the workman to his employment after serious injury. The address was most interesting and was illustrated by a film. A vote of thanks for the presidential address was proposed by Mr. Jackman and seconded by Dr. Richard Clarke, and carried with acclamation.

4. Annual reports of the Secretary and Treasurer were then read.

5. The Treasurer revealed a balance of about £800 in hand' although the last year's expenditure had exceeded receipts by nearly £400. This was, however, accounted for by the several non-recurring items, including £250 sent to the Medical War Relief Fund. Two appeals for financial assistance from the Society were referred to the Committee for their recommendation.

6. The Editor of the *Journal*, Dr. Nixon, gave his report, which was accepted (see pp. 155–6).

7. Election of Officers for the ensuing year :—

The *Honorary Secretary*, Mr. A. L. Eyre-Brook, and the *Honorary Treasurer*, Dr. Sutton, were re-elected to serve a further year.

*Medical Librarians.*—It was suggested that the Professors of Medicine, Obstetrics and Pathology should serve again, and that they should have power to co-opt Professor Milnes Walker, who had already been proposed for membership.

*Members on the University Library Sub-Committee.*—It was suggested from the Chair that the three Clinical Professors should be asked to watch the interests of the Society on this Sub-Committee. This was approved.

*The Honorary Treasurer's Report will appear in next issue.*

---

**The Programme for the 1946–47 Session.**

FEBRUARY.—Professor H. J. Seddon will give a paper on "**The Infantile Paralysis Epidemic in Malta.**"

During the three remaining meetings, we are hoping to have an address from a guest speaker on some pediatric problem, a paper on the "**Rhesus Factor**" by Dr. Beryl Corner and Dr. Tovey, and an evening devoted to short papers.

**Editor's Report**

SINCE the beginning of 1940, issues of the *Journal* have been as follows :—

| | | | | |
|---|---|---|---|---|
| 1940 | ... | Nos. 215 Spring | 216 Summer | 217 Winter |
| 1941 | ... | Nos. 218 Spring | 219 Autumn | |
| 1942 | ... | Nos. 220 Spring | 221 Autumn | |
| 1943 | ... | No. 222 Autumn | | |
| 1944 | ... | No. 223 Summer | | |
| 1945 | ... | No. 224 Autumn | | |
| 1946 | ... | Nos. 225 Spring | 226 Summer | 227 Autumn |

It must be remembered that the *Journal* was founded and exists for publishing the Transactions of the Society. Since meetings of the Society were suspended during the war there were no Transactions to publish and, in consequence, the issues of the *Journal* were greatly reduced. With restrictions on paper, the size of the *Journal* was cut down and it was printed in a smaller type.

The reduction in the number of issues and the size of each *Journal* has cut down the cost of the *Journal* as a whole. But printing and publishing costs have risen and if the Society wishes to continue publishing the *Journal* quarterly, the cost of production will have to be met. I estimate that if we are content with a 52-page *Journal*, printed in smaller type (as at present), the cost of each number will be about £50. Advertisements pay part. If we raise the price of the *Journal* from 3s. to 4s. per copy, I think it will be possible to continue without raising the Annual Subscription to the Society. At present the *Journal* receives 10s. 6d. per annum from each member's subscription of 21s. If the amount allotted can be increased to 16s., there will remain 5s. of the subscription for the other expenses of the Society.

The Treasurer believes this will be sufficient for our present expenses.

Non-members will be asked to pay 16s. instead of 10s. 6d. for the four copies per year.

As regards illustrations, in future, I am afraid, we must revert to our former practice of only publishing illustrations at the author's expense.

### HOW THE "JOURNAL" IS FINANCED.

During the years 1939–46 the number of members of the Society was as follows: 1939–40, 267; 1941, 267; 1942, 245; 1943, 245; 1944, 232; 1945, 224; 1946, 255; exclusive of Honorary and Service Members. For the first and last years of that period the subscription was 10s. 6d.; for the other years 9s. yearly. The amount due from the Society for *Journals* supplied to members is, therefore :—

| | £ | s. | d. |
|---|---|---|---|
| 522 subscriptions at 10s. 6d | 274 | 1 | 0 |
| 1,213 subscriptions at 9s. ... | 545 | 17 | 0 |
| | £819 | 18 | 0 |

**1939-46**

| EXPENDITURE. | £ | s. | d. | RECEIPTS. | £ | s. | d. |
|---|---|---|---|---|---|---|---|
| J. W. Arrowsmith, printing, etc. .. | 762 | 15 | 5 | By Non-Members Subscriptions, Payments for Reprints, etc. .. | 43 | 11 | 6 |
| Postages and Sundries .. .. | 9 | 15 | 0 | By Payment from Society on a/c Subscriptions Due .. .. | *635 | 0 | 0 |
| Audit Fee (7 years) .. .. | 3 | 13 | 6 | Balance .. .. .. .. | 97 | 12 | [illegible] |
| | £776 | 3 | 11 | | £776 | 3 | 11 |

* NOTE.—The Society's Accounts shewn on page 49 are for seven years ended September 1945, and therefore do not correspond exactly with the period of the *Journal's* accounts.

| LIABILITIES. | £ | s. | d. | £ | s. | d. | ASSETS. | £ | s. | d. | £ | s. | d. |
|---|---|---|---|---|---|---|---|---|---|---|---|---|---|
| Sundry Creditors .. | | | | 3 | 13 | 6 | Due from Society for Members' Subscriptions .. | 819 | 18 | 0 | | | |
| Balance at 30th Sept., 1939 .. .. | 105 | 7 | 4 | | | | Paid on Account .. | 635 | 0 | 0 | | | |
| Profit on 7 years .. | 87 | 5 | 7 | | | | | | | | 184 | 18 | 5 |
| Balance at 30th Sept., 1946 .. .. | | | | 192 | 12 | 11 | Cash at Bank .. | | | | 11 | 8 | [illegible] |
| | | | | £196 | 6 | 5 | | | | | £196 | 6 | 5 |

The price of books reviewed by the *Journal* and placed in the Library for the use of members during the seven years is £132 16s. 2d.: this does not include British and Foreign Journals received in exchange for the Society's *Journal*, 103 in 1939 and 70 in 1946.

